# Dysregulated Zn^2+^ homeostasis impairs cardiac type-2 ryanodine receptor and mitsugumin 23 functions, leading to sarcoplasmic reticulum Ca^2+^ leakage

**DOI:** 10.1074/jbc.M117.781708

**Published:** 2017-06-19

**Authors:** Benedict Reilly-O'Donnell, Gavin B. Robertson, Angela Karumbi, Connor McIntyre, Wojciech Bal, Miyuki Nishi, Hiroshi Takeshima, Alan J. Stewart, Samantha J. Pitt

**Affiliations:** From the ‡ School of Medicine, University of St. Andrews, St. Andrews, KY16 9TF, Scotland, United Kingdom,; § Department of Biophysics, Institute of Biochemistry and Biophysics, Polish Academy of Science, Warsaw, 02-106 Poland, and; ¶ Graduate School of Pharmaceutical Sciences, Kyoto University, Kyoto 606-8501, Japan

**Keywords:** calcium, excitation-contraction coupling (E-C coupling), heart failure, ion channel, ryanodine receptor, zinc, MG23

## Abstract

Aberrant Zn^2+^ homeostasis is associated with dysregulated intracellular Ca^2+^ release, resulting in chronic heart failure. In the failing heart a small population of cardiac ryanodine receptors (RyR2) displays sub-conductance-state gating leading to Ca^2+^ leakage from sarcoplasmic reticulum (SR) stores, which impairs cardiac contractility. Previous evidence suggests contribution of RyR2-independent Ca^2+^ leakage through an uncharacterized mechanism. We sought to examine the role of Zn^2+^ in shaping intracellular Ca^2+^ release in cardiac muscle. Cardiac SR vesicles prepared from sheep or mouse ventricular tissue were incorporated into phospholipid bilayers under voltage-clamp conditions, and the direct action of Zn^2+^ on RyR2 channel function was examined. Under diastolic conditions, the addition of pathophysiological concentrations of Zn^2+^ (≥2 nm) caused dysregulated RyR2-channel openings. Our data also revealed that RyR2 channels are not the only SR Ca^2+^-permeable channels regulated by Zn^2+^. Elevating the cytosolic Zn^2+^ concentration to 1 nm increased the activity of the transmembrane protein mitsugumin 23 (MG23). The current amplitude of the MG23 full-open state was consistent with that previously reported for RyR2 sub-conductance gating, suggesting that in heart failure in which Zn^2+^ levels are elevated, RyR2 channels do not gate in a sub-conductance state, but rather MG23-gating becomes more apparent. We also show that in H9C2 cells exposed to ischemic conditions, intracellular Zn^2+^ levels are elevated, coinciding with increased MG23 expression. In conclusion, these data suggest that dysregulated Zn^2+^ homeostasis alters the function of both RyR2 and MG23 and that both ion channels play a key role in diastolic SR Ca^2+^ leakage.

## Introduction

During the cardiac cycle, myocardial contraction is initiated when calcium ions (Ca^2+^) influx into the cell and bind to and activate the type-2 ryanodine receptors (RyR2)^4^ located within the dyad. The opening of RyR2 channels cause the release of Ca^2+^ from the sarcoplasmic reticulum (SR), which results in a transient rise in cytosolic Ca^2+^. Relaxation of cardiac muscle follows when the concentration of Ca^2+^ is sufficiently reduced due to the combined action of RyR2 channel closure, extrusion of Ca^2+^ from the cell, and uptake of Ca^2+^ back into the SR Ca^2+^ stores. In addition to Ca^2+^, the activity of RyR2 is in part regulated by Mg^2+^, which helps prevent inappropriate channel openings during diastole (1). Given the essential role of RyR2 channels in excitation-contraction (EC) coupling, it is unsurprising that these channels are key players in heart failure and fatal arrhythmias, where damaging changes to Ca^2+^ homeostasis occurs.

In heart failure it is thought that RyR2 channels become abnormally active or “leaky” and are unable to remain closed during diastole (2, 3). This leads to an increase in spontaneous Ca^2+^ spark frequency and dysregulated Ca^2+^ handling within the cardiomyocyte, resulting in decreased systolic contraction and irregular contractile activity (4, 5). In addition to inappropriate activity, it has been reported that a subpopulation of RyR2 channels preferentially gate in a long-lived sub-conductance state within the failing heart (2). Although it is undisputed that RyR2 channels contribute directly to the pathology of heart failure, the underlying cause of abnormal RyR2 functioning remains uncertain. There is also evidence for an RyR2-independent mechanism of SR Ca^2+^ efflux and that these ionic fluxes are more strongly activated in disease states (6). The identity of this alternative leak channel is currently unknown.

Mitsugumin 23 (MG23) is a recently identified voltage-dependent non-selective cation-conducting channel shown to have particular abundance on the endoplasmic/sarcoplasmic reticulum (ER/SR) membrane (7, 8). Very little is known regarding the physiological role and function of MG23 but given the location of MG23 together with the demonstration that it is permeable to Ca^2+^, it has been suggested that it may play a role alongside RyR2 in shaping intracellular Ca^2+^-dynamics and be important in SR Ca^2+^ leak (8, 9).

In chronic heart failure, aberrant Zn^2+^ homeostasis is associated with dysregulated intracellular Ca^2+^ release, reduced cardiac contractility, and significantly prolonged elevations of systolic Ca^2+^ (10–12). This suggests a key role for Zn^2+^ in regulating cardiac function. Cardiomyocytes contain a small but measurable pool of free Zn^2+^ in the cytosol, which is reported to be ∼100 pm (13). This basal level of Zn^2+^ is transiently altered during cardiac EC-coupling as a result of both the influx of Zn^2+^ into the cell through L-type Ca^2+^ channels and the release of Zn^2+^ from intracellular stores including the ER/SR (14). We recently showed that RyR2-mediated Ca^2+^-homeostasis is intimately related to intracellular Zn^2+^ levels and suggest that physiological levels of Zn^2+^ are essential in fine-tuning the release of Ca^2+^ from the SR during cardiac EC coupling (15). We also showed that when Zn^2+^ levels reach ≥1 nm, Zn^2+^ directly activates RyR2 channels, and the dependence of Ca^2+^ for channel activation is removed.

The role of Zn^2+^ in regulating diastolic Ca^2+^ leak is poorly characterized. Here we examine the role of Zn^2+^ in shaping SR Ca^2+^ release. We propose that Zn^2+^ alters the gating of both RyR2 and MG23 and that this is likely to play a key role in diastolic Ca^2+^ leak leading to the progression of heart failure and the generation of fatal arrhythmias.

## Results

### Zn^2+^ increased RyR2 activity in the presence of the endogenous inhibitor Mg^2+^

The activity of RyR2 is known to depend on its interactions and regulation by multiple endogenous effector molecules including Ca^2+^ and Mg^2+^ (1, 16–18). Inhibition of RyR2 by Mg^2+^ helps prevent activation of RyR2 during diastole (19–21). The mechanism by which RyR2 remains sensitive to Ca^2+^ even in the presence of cytosolic free Mg^2+^ is unknown. Physiological concentrations of Mg^2+^ are thought to be within the millimolar range (∼1 mm). Interestingly, at this concentration, cardiac RyR2 channels incorporated into planar bilayers respond poorly to systolic concentrations of Ca^2+^ (22, 23). In the failing heart, RyR2 sensitivity to inhibition by cytosolic Mg^2+^ is reduced when compared with healthy controls (24).

**Figure 1.**
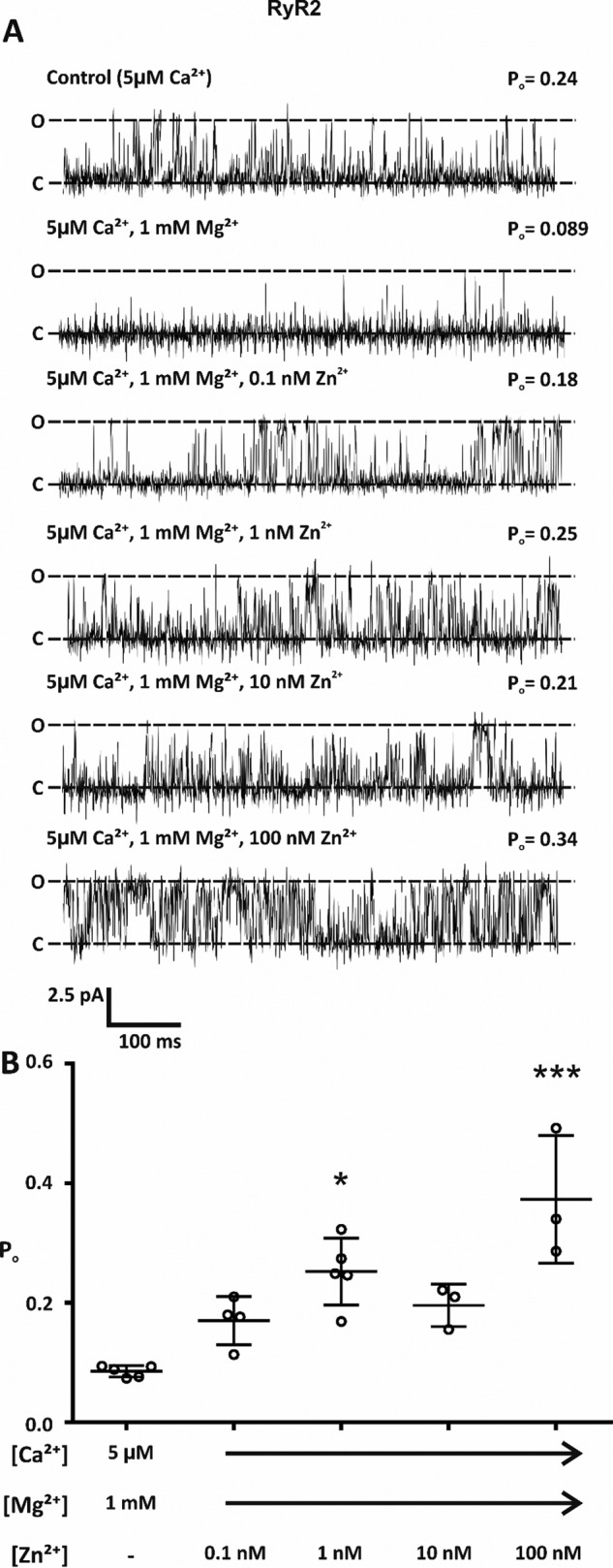
**Cytosolic Zn^2+^ increased RyR2 activity in the presence of 1 mm Mg^2+^.** *A*, representative single RyR2 channel traces with Ca^2+^ as the permeant ion. Bilayers were voltage-clamped at 0 mV. *O* and *C* show the fully open and closed states, respectively. In the presence of activating levels of cytosolic Ca^2+^ (5 μm), the addition of 1 mm Mg^2+^ reduced RyR2 activity. In the continued presence of 1 mm Mg^2+^, the subsequent addition of Zn^2+^ to the cytosolic face of the channel increased channel open probability. *B*, scatter plot showing the mean channel open probability of RyR2. Individual data points are shown by ○. Data are expressed as the mean ± S.D.; *n* = 3–5. Significant difference between treatments was assessed by a one-way ANOVA; (F(5,18) = 7.276, *p* = 0.0007) followed by a Bonferroni post hoc test. * (*p* < 0.05) and *** (*p* < 0.001) indicate significance when compared with Mg^2+^-treated control.

To determine if Zn^2+^ plays a key role in relieving RyR2 inhibition by Mg^2+^, single RyR2 channels were incorporated into phospholipid bilayers and the direct action of Zn^2+^ at the cytosolic face of the channel in the presence of 1 mm Mg^2+^ and activating levels of Ca^2+^ (5 μm) was studied (Fig. 1). Using Ca^2+^ as the permeant ion and holding at a command potential of 0 mV, in line with previous studies (16, 17), the addition of 1 mm Mg^2+^ significantly reduced channel open probability (*P*_o_) from 0.26 ± 0.13 to 0.09 ± 0.01 (Fig. 1*A*, *p* = 0.017; *n* = 4–5). In the continued presence of 1 mm Mg^2+^ the subsequent addition of 0.1 nm Zn^2+^ to the cytosolic face of the channel caused channels to gate with a comparable *P*_o_ to Mg^2+^-free Ca^2+^-activated control channels (0.17 ± 0.04 compared with 0.26 ± 0.13; *n* = 4). This suggests that Zn^2+^ plays a key role in regulating channel function enabling RyR2 to operate under conditions of systole. When Zn^2+^ levels were further incremented in the range 0.1-100 nm, channel activity was significantly increased from Mg^2+^-treated control channels (Fig. 1, *A* and *B*). However, there was no significant difference between individual Zn^2+^ treatments (Fig. 1*B*).

### Pathophysiological concentrations of Zn^2+^ led to leaky RyR2 channels

**Figure 2.**
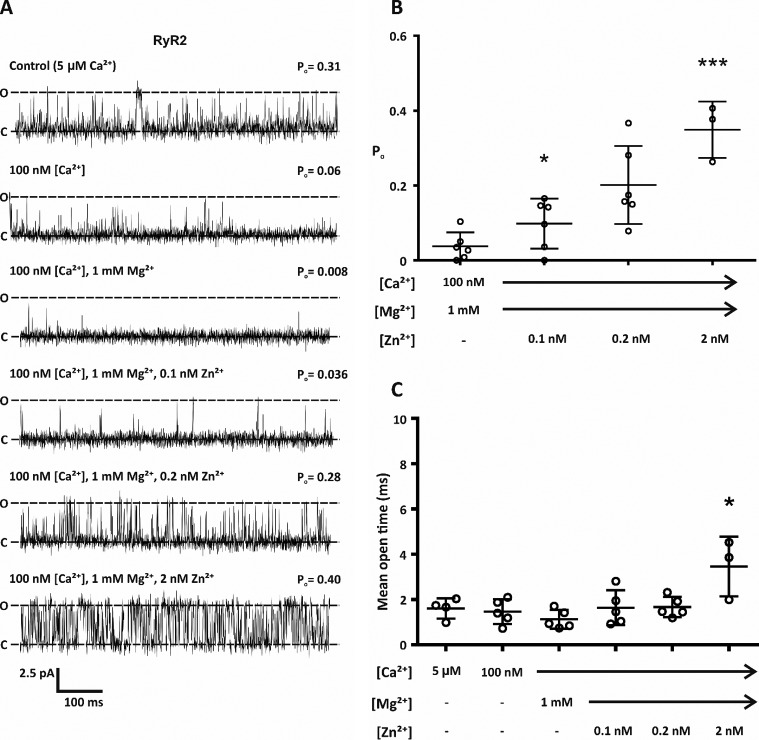
**Under diastolic conditions, pathophysiological levels of cytosolic free Zn^2+^ resulted in increased RyR2 activity.** *A*, representative single RyR2 channel traces. Bilayers were voltage-clamped at 0 mV, and Ca^2+^ was the permeant ion. Open and closed states of RyR2 are indicated by *O* and *C*. As expected, RyR2 activity was reduced when the cytosolic Ca^2+^ were lowered from 5 μm to 100 nm and reduced further after the addition of 1 mm Mg^2+^. The addition of 2 nm Zn^2+^ to the *cis* chamber significantly increased channel activity. *B*, mean open probability of RyR2. Individual data points are shown by ○. Data are shown as the mean ± S.D.; *n* = 3–5. The difference between treatments was assessed by a one-way ANOVA followed by a Bonferroni post hoc test. F(5,21) = 8.133; *p* = 0.0002. * (*p* < 0.05) and *** (*p* < 0.001) indicate significance when compared with Mg^2+^-treated channels in the presence of 100 nm Ca^2+^. *C*, scatter plot showing the mean channel open time. Data are shown as the mean ± S.D.; *n* = 3–5. Individual data points are shown by ○. One-way ANOVA followed by a Bonferroni test was used to test difference between treatments (F(5,26) = 8.501, *p* < 0.0001). * indicates significance (*p* ≤ 0.05) compared with Mg^2+^-treated channels in the presence of 100 nm Ca^2+^.

In heart failure RyR2 channels become inappropriately activated during diastole. Because levels of Zn^2+^ are altered in heart failure and can be chronically elevated by as much as 30-fold (25), we next wanted to investigate whether Zn^2+^ could modulate RyR2 function in the presence of diastolic concentrations of Ca^2+^ (100 nm) and in the continued presence of 1 mm Mg^2+^. Using Ca^2+^ as the permeant ion and holding at a command potential of 0 mV, when the cytosolic free Ca^2+^ was reduced from 5 μm to 100 nm, as expected, channel *P*_o_ was reduced (Fig. 2*A*). Consistent with the role of Mg^2+^ as an endogenous regulator of channel activity, the addition of 1 mm Mg^2+^ to the cytosolic face of the channel resulted in a further reduction in channel *P*_o_ (Fig. 2*A*). Under these conditions nearly all channel openings were abolished (Fig. 2*B*). The addition of physiological concentrations of Zn^2+^ (0.1 nm) did not significantly increase channel activity (Fig. 2, *A* and *B*). However, elevation of free Zn^2+^ to pathophysiological levels (∼2 nm) resulted in a significant increase in channel *P*_o_ (Fig. 2*B*).

We next looked at the mean open time of RyR2 gating after the addition of Zn^2+^ under diastolic conditions. Channel gating after the addition of Zn^2+^ ≤0.2 nm was not significantly different compared with openings from Ca^2+^-activated control channels (Fig. 2*C*), suggesting that in the presence of physiological levels of free Zn^2+^, channels are still regulated by Ca^2+^. Importantly, elevating the free Zn^2+^ concentration to 2 nm caused channels to gate to a comparable *P*_o_ to those activated by systolic levels of Ca^2+^ (*P*_o_ values were 0.35 ± 0.08 and 0.26 ± 0.09; *n* = 3–6), but the mode of gating under these conditions was altered (Fig. 2*C*). Under these conditions, channels gated in significantly longer-lived open states compared with control. This type of gating is consistent with Zn^2+^-dependent openings.

### RyR2 channels were not the only sarcoplasmic reticulum Ca^2+^-permeable channels regulated by Zn^2+^

**Figure 3.**
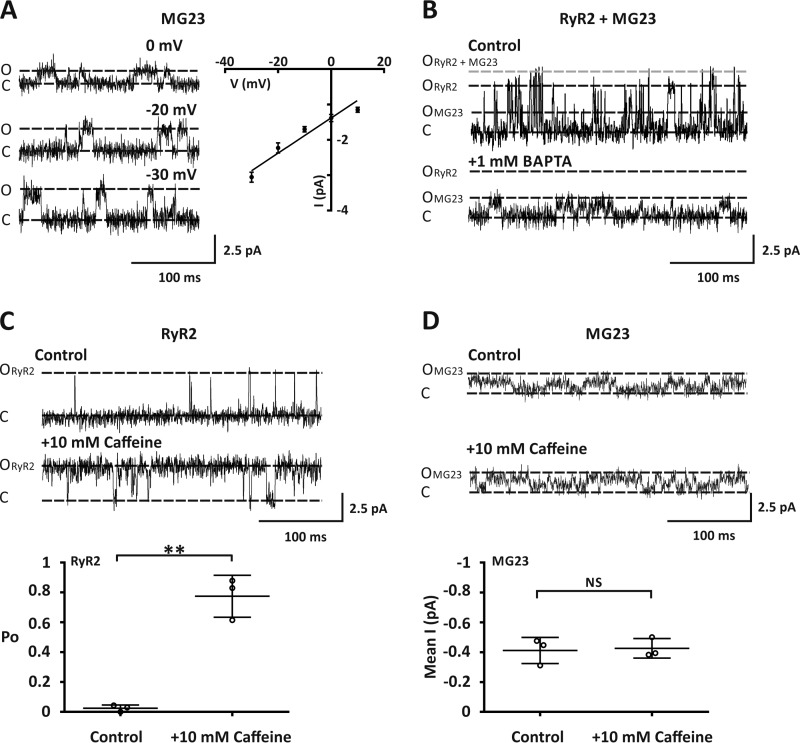
**Native cardiac MG23 channels displayed distinct gating properties from RyR2.** *A*, the *left panel* shows representative Ca^2+^ current fluctuations mediated by MG23, recorded at various holding potentials. The traces were chosen to show current amplitudes clearly. The *right panel* shows a current-voltage relationship for MG23 using Ca^2+^ as a permeant ion. Data are shown as the mean ± S.D. (*n* = 5). *B*, representative current fluctuations through both MG23 and RyR2 channel gating in the same bilayer under control conditions with Ca^2+^ as a permeant ion at a holding potential of 0 mV. Cytosolic free Ca^2+^ was 5 μm. MG23 (*O_MG23_*), RyR2 (*O_RyR2_*) open, RyR2 + MG23 (*O*_*RyR2*+*MG23*_) open and closed (*C*) states are indicated. The addition of 1 mm BAPTA to the *cis* chamber lowers Ca^2+^ to <4 nm. This Ca^2+^ concentration was sub-activating for RyR2 but had no effect on MG23. *C*, the *top panel* shows representative Ca^2+^ current fluctuations mediated through RyR2 under control conditions with Ca^2+^ as the permeant ion. Cytosolic-free Ca^2+^ was 5 μm. The addition of 10 mm caffeine to the *cis* chamber resulted in activation of RyR2 as expected. The *lower panel* shows a scatter plot of RyR2-channel open probability under these conditions. Data are shown as the mean ± S.D.; *n* = 3. ** denotes a significant difference (*p* < 0.01) to control. *D*, the *top panel* shows representative Ca^2+^ current fluctuations mediated through MG23 under control conditions using Ca^2+^ as the permeant ion. Cytosolic free Ca^2+^ was 5 μm. The addition of 10 mm caffeine to the *cis* chamber had no effect on MG23 channel activity. The *lower panel* shows a scatter plot of mean average current mediated through MG23 under these conditions. Data are shown as the mean ± S.D.; *n* = 3.

In experiments where Ca^2+^ currents were observed, 38% (15/39) of our bilayers displayed openings that were not characteristic of RyR2 gating (Fig. 3*A*). Using Ca^2+^ as the permeant ion, construction of a current-voltage relationship revealed that this other SR Ca^2+^-permeable channel displayed a Ca^2+^ conductance of 51 ± 1.5 picosiemens (Fig. 3*A*). This is consistent with the single channel Ca^2+^ conductance reported previously for the SR ion-channel MG23 (8). In 31% of our bilayers (12/39) where Ca^2+^ currents were observed, we detected gating from both RyR2 and MG23 (Fig. 3*B*). In these experiments, reducing the level of Ca^2+^ to a subactivating concentration (4 nm) by the addition of 1 mm 2,2′-ethylenedioxy)dianiline-*N,N,N*′*,N*′-tetraacetic acid (BAPTA) abolished RyR2 openings as expected, but MG23 openings were still apparent. In the presence of activating levels of Ca^2+^ (5 μm), the addition of 10 mm caffeine, which is a known activator of RyR2 (26), significantly increased RyR2 activity (Fig. 3*C*). It has been shown previously that MG23 openings are very fast and frequent and that multiple channels often gate together in the bilayer in a coordinated manner (8). We also observed this type of gating when MG23 was incorporated into the bilayer. To assess channel activity, we therefore, used noise analysis. When MG23 was gating in the bilayer, the addition of 10 mm caffeine had no effect on channel activity (Fig. 3*D*). This reveals that the regulation of MG23 and RyR2 is markedly different.

### Zn^2+^ regulated the activity of purified MG23

**Figure 4.**
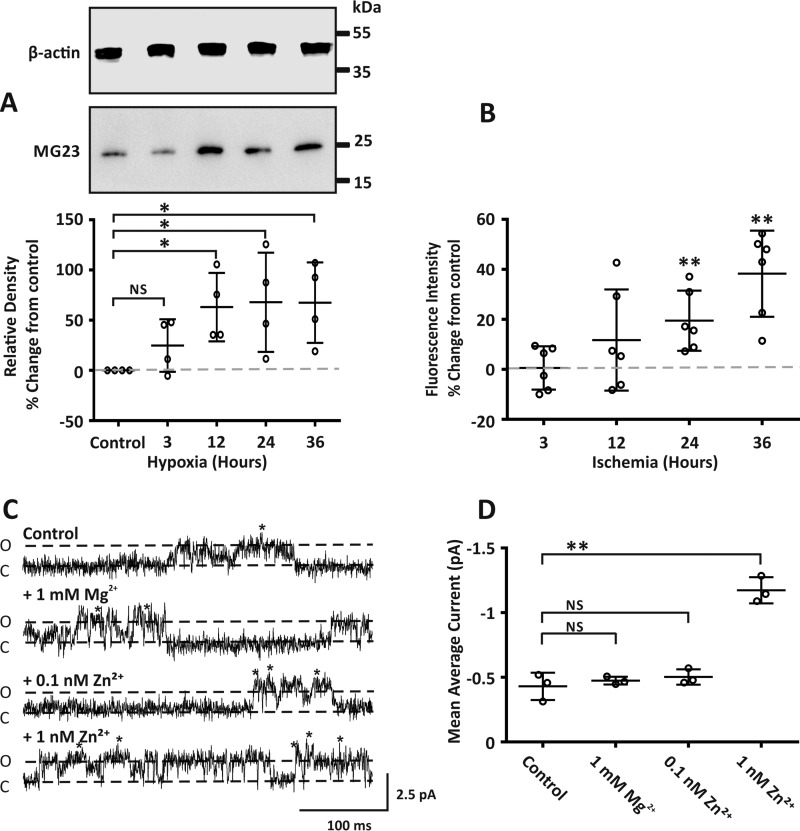
**Pathophysiological concentrations of Zn^2+^ altered both the expression and activity of MG23.** *A*, the *upper panel* representative Western blot shows MG23 protein expression in H9C2 cells exposed to hypoxia. All lanes were loaded with 10 μg of total protein. Western blots were probed with antibodies specific for MG23 or with β-actin as a loading control. Size markers are indicated in kDa. The *lower panel* scatter plot shows relative density of MG23 under labeled conditions. Band densities were normalized to β-actin loading controls in each lane and are expressed relative to control as % percentage change. The *gray dashed line* denotes control values. Data are displayed as the mean ± S.D. (*n* = 4). * denotes a significant difference (*p* < 0.05) to control. *B*, scatter plot showing ZnAF-2 DA fluorescence intensity from H9C2 cells exposed to ischemia buffer for indicated time points. For each time point ZnAF-2 DA fluorescence was normalized to DAPI nuclear staining fluorescence intensity for each corresponding well and expressed as percentage change from control. Control values are indicated by the *gray dotted line*. Data are displayed as the mean ± S.D. (*n* = 6) from two individual cell preparations. ** denotes a significant difference (*p* < 0.01) to control. *C*, MG23 single-current fluctuations show increased channel activity with increasing concentrations of cytosolic Zn^2+^, in the presence of physiological levels (1 mm) Mg^2+^. The traces were chosen to show clear channel openings. Cytosolic free Ca^2+^ was 5 μm. Open (*O*) and closed (*C*) states are indicated. Holding potential was 0 mV, and Ca^2+^ was used as the permeant ion. Multiple MG23 often incorporated into the bilayer, indicated by *. *D*, scatter plot showing the mean average current under conditions described in *C*. Data are displayed as the mean ± S.D. (*n* = 3). ** indicates a significant difference (*p* < 0.01) to control. *NS*, not significant.

The role of RyR2 in SR Ca^2+^ leak in heart failure is widely accepted. Here we investigate if MG23 also contributes to the release of Ca^2+^ from SR stores alongside RyR2. In heart failure, cardiac cells encounter hypoxic conditions (27). We, therefore, investigated if the protein expression of MG23 was altered under conditions of hypoxia. Using the cardiac cell line H9C2, Western blot analysis revealed that the protein level of MG23 increased incrementally after periods (3–36 h) of hypoxia (Fig. 4*A*). Using the Zn^2+^-specific fluorescent indicator ZnAF-2 diacetate (DA), we measured the relative cumulative fluorescent intensity and show that intracellular Zn^2+^ levels in H9C2 cells are elevated when exposed to ischemic conditions (Fig. 4*B*). This is important as dysregulated Zn^2+^ homeostasis is linked with ischemia and chronic heart failure (10, 11, 28–30).

We next assessed if Zn^2+^ could directly modulate the activity of MG23. MG23 was purified from sheep cardiac ventricular tissue following previously established methods (8). Construction of a current-voltage relationship for purified MG23 revealed a single channel Ca^2+^ conductance of 49 ± 0.9 picosiemens (supplemental Fig. S1). Using Ca^2+^ as the permeant ion, in the presence of physiological levels of Mg^2+^ (1 mm) and holding at a command potential of 0 mV, the addition of 0.1 nm Zn^2+^ to the cytosolic face of MG23 had no significant effect on channel activity (Fig. 4, *C* and *D*). Interestingly a 10-fold increase in the concentration of Zn^2+^ from 0.1 nm to 1 nm had a significant effect on MG23 channel activity (Fig. 4*D*).

### Zn^2+^ does not cause RyR2 sub-conductance-state gating

**Figure 5.**
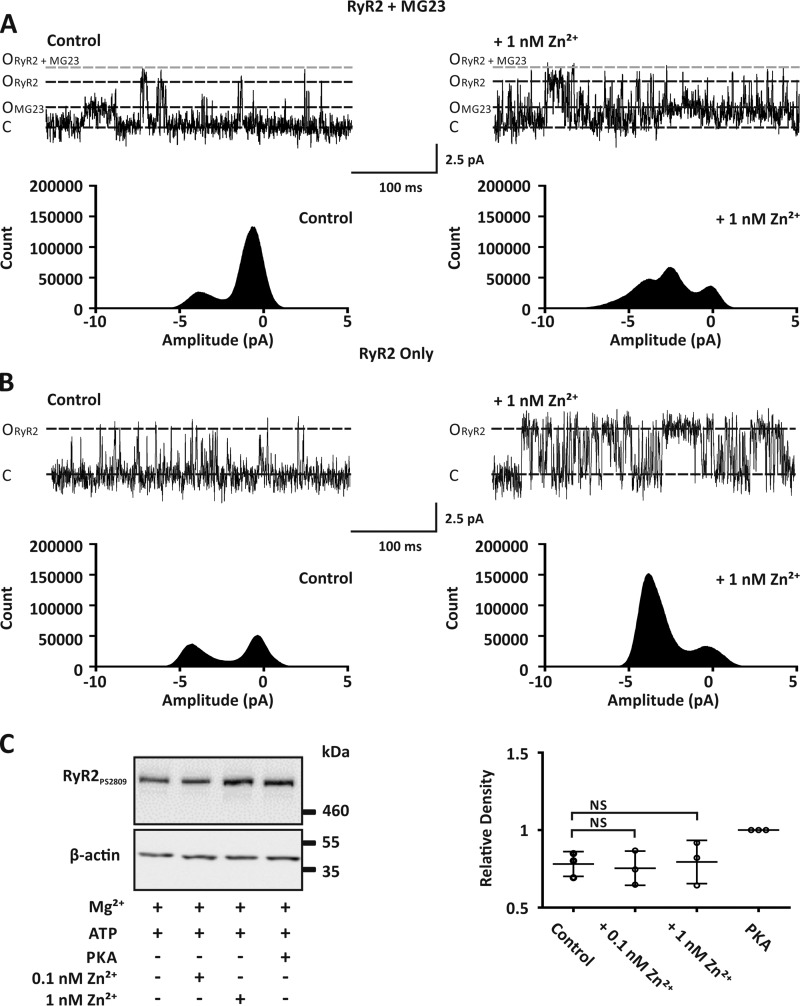
**Zn^2+^ modulated the activity of both MG23 and RyR2 but did not result in RyR2 sub-conductance gating.** *A*, representative Ca^2+^ current fluctuations when both MG23 and RyR2 channels were gating in the same bilayer (*left trace*) and after the subsequent addition of 1 nm Zn^2+^ to the *cis* chamber (*right trace*). Cytosolic free Ca^2+^ was 5 μm. MG23 (*O_MG23_*), RyR2 (*O_RyR2_*) open, RyR2 + MG23 (*O*_*RyR2*+*MG23*_) open and closed (*C*) states are indicated. The corresponding all-points-amplitude histogram is shown *underneath* the respective single-channel recording. Recordings were made using Ca^2+^ as the permeant ion and at a holding potential of 0 mV. *B*, representative Ca^2+^ current fluctuations through RyR2 channels only (*left trace*) and after the subsequent addition of 1 nm cytosolic Zn^2+^ (*right trace*). Cytosolic free Ca^2+^ was 5 μm. Corresponding all-points-amplitude histograms are shown *underneath* the respective single-channel recording. *C*, *left panel*, Western blot analysis showing the phosphorylation of RyR2 at site serine 2809 after incubation of cardiac HSR vesicles with either 1 unit/μg of protein of the PKA active subunit, 0.1 nm Zn^2+^, or 1 nm Zn^2+^. In all experiments 1 mm Mg^2+^ and 1 mm ATP were present. All lanes were loaded at 30 μg of total protein. Western blots were probed with antibodies specific for RyR2 phosphorylated at site serine 2809 or with β-actin as a loading control. Size markers are indicated in kDa. The *right panel* scatter plot shows relative density of RyR2 phosphorylation levels under these conditions. Band densities were normalized to β-actin loading controls in each lane and expressed relative to phosphorylation by PKA. Data are displayed as the mean ± S.D. (*n* = 3).

We next wanted to investigate if native MG23 channels provide a leak Ca^2+^ current in response to elevated Zn^2+^ levels when gating together with RyR2. In control recordings where both RyR2 and MG23 were gating, using Ca^2+^ as the permeant ion and holding at a command potential of 0 mV, construction of an all-points histogram from our single-channel data displayed two clear peaks (Fig. 5*A*, *left*). In control recordings, amplitude histograms were best fit with a mixture of two Gaussian distributions displaying mean values of −0.47 ± 0.16 pA and −4.03 ± 0.23 pA (Table 1). These values represent the closed and the full open state of RyR2, respectively. Under these conditions RyR2 activity is low, and the number of events for the closed state is larger than the number of events for the open state. The contribution from MG23 is negligible. The subsequent addition of 1 nm Zn^2+^ to the *cis* chamber not only increased RyR2 channel *P*_o_ but also revealed a third peak (Fig. 5*A*, *right*). Amplitude histograms were now best fit with a mixture of three Gaussian distributions (Table 1). The smaller open state (−2 ± 0.5 pA) we now reveal is within the range of the unitary current amplitude that we (Fig. 3*A*) and others (8) report for native MG23 channels when voltage-clamped at 0 mV. In bilayers where only RyR2 is gating, as expected the addition of 1 nm Zn^2+^ increased RyR2 activity and more events resided in the open state (Fig. 5*B*). However, only two peaks were ever observed, and data were best fit with a mixture of two Gaussian distributions (Table 1), as there is no contribution from MG23 (Fig. 5*B*, *right*).

**Table 1:** **MG23 gating is revealed by pathophysiological concentrations Zn^2+^** Shown are the mean Gaussian fits of paired all-points-amplitude histograms constructed from bilayer experiments using native SR vesicle preparations in the absence and presence of 1 nm Zn^2+^. Bilayers were voltage-clamped at 0 mV, and Ca^2+^ was the permeant ion. Data are displayed as the mean ± S.D., *n* = 3.

Experiment number	Gaussian fits of all-points-amplitude histograms					
	Control			1 nm Zn^2+^		
	Peak 1	Peak 2	Peak 3	Peak 1	Peak 2	Peak 3
	*pA*			*pA*		
**Bilayers containing RyR2 and MG23**						
1	−0.65	−3.78		−0.167	−2.53	−3.76
2	−0.39	−4.24		−0.489	−1.62	−4.41
3	−0.36	−4.07		0.3	−1.87	−4.4
Mean ± S.D.	−0.47 ± 0.2	−4.03 ± 0.2		−0.12 ± 0.4	−2 ± 0.5	−4.19 ± 0.4

**Bilayers containing RyR2 only**						
1	−0.38	−4.22		−0.41	−3.8	
2	−0.25	−3.88		−0.04	−4.22	
3	0.06			−0.31	−4.14	
Mean ± S.D.	−0.19 ± 0.2	−4.05 ± 0.2		−0.25 ± 0.2	−4.05 ± 0.2	

Previous studies have suggested that in heart failure RyR2 becomes hyperphosphorylated at serine 2808/2809, which leads to the dissociation of the FK-506-binding protein 1B (FKBP12.6), resulting in sub-state gating (2). We next wanted to assess if Zn^2+^ altered the phosphorylation status of RyR2 at site serine 2809. Incubation of isolated SR vesicles with 1 nm Zn^2+^ had no effect on the level of phosphorylation at site serine 2809 (Fig. 5*C*).

**Figure 6.**
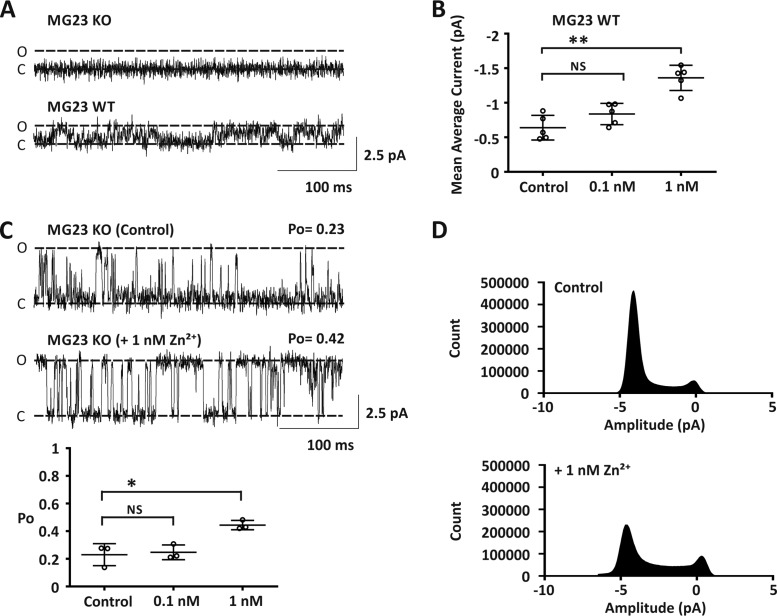
**Zn^2+^ potentiated RyR2 activity in *Mg23* knock-out mice.** *A*, representative Ca^2+^ current fluctuations from bilayer experiments using cardiac SR vesicles isolated from wild-type and *Mg23*-knock-out mice. Functional MG23 channel gating was observed in wild-type experiments but not in *Mg23* knock-out experiments. Open (*O*) and closed (*C*) states are indicated. Holding potential was 0 mV, and Ca^2+^ was used as the permeant ion. Cytosolic free Ca^2+^ was 5 μm. *B*, scatter plot showing noise analysis data from wild-type experiments where only MG23-mediated Ca^2+^ currents were observed. Mean average current is shown after incremental addition of Zn^2+^ to the *cis* chamber. *C*, representative Ca^2+^ current fluctuations from bilayer experiments using cardiac SR vesicles isolated from *Mg*23 knock-out mice showing functional RyR2 channel gating in the absence and presence of 1 nm Zn^2+^ added to the *cis* chamber. The *lower panel* scatter plot shows RyR2 channel open probability under these conditions. *D*, paired all-points-amplitude histograms under conditions described in *C*. Data shown are the mean ± S.D., *n* = 3, with * and ** indicating significant differences (*p* < 0.05 and *p* < 0.01), respectively, from control. *NS*, not significant.

To confirm that Zn^2+^ does not result in RyR2 sub-conductance-state gating, we prepared SR vesicles from *Mg23* knock-out mice. This allowed us to investigate RyR2-mediated Ca^2+^ currents in isolation from MG23. In these experiments we never observed gating from MG23 (*n* = 18; Fig. 6*A*, *upper trace*). In contrast, MG23-mediated Ca^2+^ currents were observed in 22% (*n* = 5/23) of our bilayer experiments when we used SR vesicles prepared from wild-type mice (Fig. 6*A*, *lower trace*). In these experiments MG23 channel activity was significantly increased after the addition of 1 nm Zn^2+^ to the *cis* chamber (Fig. 6*B*). SR vesicles prepared from *Mg23* knock-out mice displayed functional RyR2 channel gating (Fig. 6*C*; *n* = 3/18), and consistent with our previous findings the addition of 1 nm Zn^2+^ to the cytosolic face of RyR2 significantly increased channel *P*_o_ (Fig. 6*C*). Construction of all-points-amplitude histograms from our single-channel data showed that the addition of 1 nm Zn^2+^ to the *cis* chamber significantly increased RyR2 activity (Fig. 6*D*). There was no evidence of RyR2 sub-conductance state gating. All data were best fit with a mixture of two Gaussian distributions representing the closed and fully open state of RyR2 (Table 2).

**Table 2:** **Fit of the all points amplitude histogram constructed from *Mg23* KO mice using Ca^2+^ as the permeantion** Shown are Mean Gaussian fits of paired all-points-amplitude histograms constructed from bilayer experiments using SR vesicles prepared from *Mg23* knockout mice in the absence and presence of 1 nm Zn^2+^. Bilayers were voltage-clamped at 0 mV, and Ca^2+^ was the permeant ion. Data are displayed as mean ± S.D., *n* = 3.

Experiment number	Gaussian fits of amplitude histograms from *mg23* KO mice			
	Control		1 nm Zn^2+^	
	Peak 1	Peak 2	Peak 1	Peak 2
	*pA*		*pA*	
1	−0.0035	−4.45	−0.15	−3.61
2	−0.53	−3.57	−0.21	−4.08
3	0.25	−4.59	−0.17	−4.09
Mean ± S.D.	−0.09 ± 0.4	−4.2 ± 0.6	−0.18 ± 0.03	−3.93 ± 0.3

## Discussion

During cardiac relaxation, it is the combined effect of both a lowered Ca^2+^ concentration (100 nm) and the presence of millimolar levels of Mg^2+^ that prevent unwanted RyR2 channel openings. This is important as even a small increase in the open probability of RyR2 during diastole will lead to a leak of Ca^2+^ into the cytoplasm, and this will have major consequences for normal cardiac function. Irregular RyR2 activity is linked to various forms of heart failure, but the underlying molecular mechanisms that cause altered RyR2 channel function remain unclear. In this study we reveal that in the presence of diastolic Ca^2+^ levels (100 nm) and 1 mm Mg^2+^, small elevations in the level of cytosolic free Zn^2+^ from resting concentrations (∼0.1 nm) to ≥1 nm results in irregular RyR2 channel openings. This provides the first evidence that dysregulated Zn^2+^ homeostasis is directly linked with leaky RyR2 channels. We also reveal that in H9C2 cardiac cells the level of intracellular Zn^2+^ becomes elevated after exposure to hypoxic conditions and that this coincides with increased protein expression of the ER/SR located non-selective cation channel MG23. Furthermore, the activity of MG23 is significantly enhanced when cytosolic Zn^2+^ is elevated from 0.1 nm to ≥1 nm. MG23 displays Ca^2+^-handling properties, suggesting that increased channel activity alongside increased channel expression may contribute to SR Ca^2+^ leak alongside RyR2.

### Zn^2+^ dyshomeostasis resulted in leaky RyR2 channels

In cardiomyocytes raised levels of intracellular Ca^2+^ result in a large release of Ca^2+^ from SR stores, and this controls contractility. In bilayer studies, however, the presence of millimolar levels of Mg^2+^ causes RyR2 channels to respond poorly to systolic Ca^2+^ levels (21, 23, 31). Under comparable experimental conditions we reveal that the addition of cytosolic Zn^2+^ in the range 0.1–100 nm increased RyR2 activity. Our data suggest that small changes in the Zn^2+^ level will have a marked effect on RyR2 activity. This becomes highly relevant when we consider that concentrations of Zn^2+^ have recently been reported to be transiently elevated to ∼50 nm during Zn^2+^-signaling events (32). Physiological levels of Zn^2+^ may, therefore, have the ability to fine-tune the activity of RyR2, suggesting a role for Zn^2+^ in regulating graded Ca^2+^-responses.

Under diastolic conditions when RyR2 channels should remain closed, the addition of 2 nm free Zn^2+^ resulted in abnormally active RyR2 channels. Under these conditions the mean open times of channel openings were significantly longer compared with channels activated by systolic levels of Ca^2+^ alone (5 μm). We recently showed that this mode of gating is consistent with Zn^2+^-dependent openings and that RyR2 is likely to have multiple Zn^2+^-binding sites (15). RyR2 is now under the control of Zn^2+^ and is decoupled from its regulatory effects of cytosolic Ca^2+^ and Mg^2+^, which impairs the ability of the RyR2 to properly deactivate, resulting in high RyR2 channel activity that may play a direct role in SR Ca^2+^ leak. In cardiac tissue, dysregulated Zn^2+^ homeostasis is linked with altered contractility and heart failure (10, 11, 29, 30, 33), but the underlying mechanisms of how Zn^2+^ contributes to these cardiomyopathies is not fully understood. Spatial imaging of trace elements reveal that in heart failure there is a significant accumulation of Zn^2+^ in the left ventricle of cardiac tissue (34). Additionally, certain cardiomyopathies including those associate with diabetes and ischemia, display chronically raised levels of intracellular Zn^2+^ (25, 28). Importantly, the accumulation of nanomolar concentrations of intracellular Zn^2+^ has been shown to be followed by a concomitant reduction in cardiomyocyte contractile function and a reduced SR calcium load (35–38), linking dysregulated intracellular Zn^2+^ levels with altered Ca^2+^ homeostasis.

Intracellular Zn^2+^ levels are regulated by ZnTs (zinc transporters) that move Zn^2+^ into organelles and out of the cell and ZIPs (Zrt/Irt-like proteins) that mediate the flux of Zn^2+^ into the cell or from organelles into the cytosol. Recent evidence links increased Zip12 expression in pulmonary vascular smooth muscle to elevated intracellular Zn^2+^ levels as a result of hypoxia, suggesting a fundamental role for Zn^2+^ in pulmonary vascular responses (39). Furthermore, increased accumulation of intracellular Zn^2+^ in isolated rat cardiomyocytes exposed to hypoxic conditions has been attributed to an imbalance in expression of ZnTs (zinc transporters) and ZIPs (30). This is highly relevant when we consider that RyRs play a key role in hypoxic increases in the intracellular Ca^2+^ concentration (40, 41) and that pulmonary hypertension is associated strongly with chronic heart failure (42, 43).

Recent evidence suggests that in heart failure SR Ca^2+^ leak causes mitochondrial calcium overload that triggers mitochondrial dysfunction and the production of free radicals, which in turn modulates RyR2 function. Reactive oxygen species induce the release of Zn^2+^ from zinc-binding metallothionein proteins (44, 45), and in cardiomyocytes, oxidants increase intracellular Zn^2+^ levels by as much as 30-fold (25). Our recent data show that concentrations of Zn^2+^ ≥1 nm directly activate RyR2, removing the dependence on Ca^2+^ for channel activation (15). The data presented in this study reveal that elevating Zn^2+^ to 1 nm even in the presence of 1 mm Mg^2+^ results in irregular channel activity. This is important as leaky RyR2 channels are considered to be the cause of mitochondrial Ca^2+^ overload and dysfunction in heart failure (46). Zn^2+^ is also reported to impair mitochondrial function, leading to the excess production of reactive oxygen species (47, 48), suggesting a potentially important role for Zn^2+^ in the feedback loop between the SR and mitochondria.

### RyR2 is not the only leaky SR Ca^2+^-permeable channel

In heart failure models RyR2 channels not only display irregular activity and are open when they should remain closed, but the reported mode of channel gating is also altered. In failing human and canine hearts, Marx *et al.* (2) reported that ∼15% of RyR2 channels gated in a long-lasting subconductance state. We show that when Zn^2+^ levels were elevated to concentrations reported to occur in chronic heart failure (>1 nm) where RyR2 and MG23 gate together, construction of an all-points amplitude histogram revealed that channel openings resided in two open states, one with a mean current amplitude consistent with the full open state of RyR2 and another with a mean current amplitude that is consistent with the unitary current amplitude of the reported RyR2 sub-conductance state. However, this smaller open state is also consistent with the mean current amplitude that we (Fig. 3*A*) and others report for MG23 at 0 mV (8). Importantly MG23 gating was never observed using SR vesicles prepared from *Mg23* knock-out mice irrespective of the Zn^2+^ concentration, whereas functional RyR2 channels were evident. In these experiments, construction of all point histograms from our single-channel data revealed that Zn^2+^ increased the activity of RyR2 but never resulted in sub-conductance-state gating. This suggests that when intracellular Zn^2+^ levels are elevated to levels similar to those in heart failure, RyR2 does not display sub-conductance gating, but rather, the activity of MG23 is increased.

Sub-conductance-state gating of RyR2 in heart failure models is thought to be a result of hyperphosphorylation of the channel leading to the dissociation of FKBP12.6. When preparing our heavy SR vesicles, any endogenous FKBPs associated with RyR2 are dissociated (49), and consistent with other groups (50–52) we do not observe high-frequency sub-state gating for RyR2.

The role of Zn^2+^ and phosphorylation at serine 2808/2809 in heart failure is unclear. Cardiomyocytes exposed to micromolar concentrations of external ZnCl_2_ display an increased level of RyR2 phosphorylation at this site (53). In contrast, phosphorylation at serine 2808/2809 is reduced when heart homogenates are incubated with micromolar levels of Zn^2+^ (12). The story is further complicated when we consider that there is controversy over the number of PKA phosphorylation sites and their associated functional effects on RyR2 gating, and perhaps more importantly, certain groups fail to observe any alteration in RyR2 phosphorylation at serine 2808/2809 in heart failure (51, 54). Phosphorylation at serine 2808/2809 has also been suggested to sensitize RyR2 to luminal Ca^2+^ (55), which is thought to lower the threshold for channel activation leading to diastolic Ca^2+^ overload and facilitating diastolic Ca^2+^ leak observed in heart failure (56). Although in this study we did not investigate the relationship between luminal Ca^2+^ and cytosolic Zn^2+^, our data show that the phosphorylation of RyR2 at serine 2808/2809 is unaltered when we incubate isolated SR vesicles with 1 nm Zn^2+^.

### Conditions of ischemic heart failure result in raised levels of intracellular Zn^2+^ and increased expression and activity of MG23

It is generally considered that the reduced SR Ca^2+^ content in heart failure is a result of reduced Ca^2+^ pumping by SERCA and increased SR Ca^2+^ leak via RyR2. We now provide evidence that MG23 contributes to SR Ca^2+^ leak alongside RyR2. Our data show that physiological resting levels of Zn^2+^ (0.1 nm) had no effect on MG23 function, but increasing the Zn^2+^ concentration to 1 nm significantly increased channel activity. Importantly we also show that intracellular Zn^2+^ levels are elevated when cardiac H9C2 cells are exposed to ischemic conditions and that this elevation in the level of Zn^2+^ coincides with an increase in the protein expression of MG23. Importantly, changes in MG23 expression in H9C2 cells after hypoxia are comparable with the reported changes in protein expression of key Ca^2+^-regulatory proteins including SERCA, phospholamban, and the sarcolemmal Na^+^/Ca^2+^ exchanger that occur in heart failure (57–59). Up-regulation of MG23 may, therefore, provide a route for increased SR Ca^2+^ release that together with the reduced expression and diminished activity of SERCA may lead to an imbalance in SR Ca^2+^ load and likely result in impaired contractility observed in heart failure. MG23 has been shown to play a role in ER-derived cell death after DNA damage (60). The increase in MG23 protein expression and consequent deleterious Ca^2+^ release may also facilitate cell death-signaling pathways in heart under pathophysiological conditions.

We propose that MG23 plays a limited role in controlling the release of Ca^2+^ under normal physiological conditions but contributes to diastolic Ca^2+^ leak in response to pathophysiological levels of Zn^2+^. This is highly relevant when we consider that there is evidence for RyR2-independent mechanisms of SR Ca^2+^ efflux and that these fluxes are strongly activated in the failing heart (6). Our data reveals that Zn^2+^ is a potent modulator of SR ion channel function and will have a major impact on the Ca^2+^-handling properties of the cell.

To conclude, our study reveals a new role for Zn^2+^ in regulating cardiac function through direct modulation of SR Ca^2+^-permeable channels. Under normal physiological conditions, we propose that intracellular Zn^2+^ plays a key role in controlling the release of Ca^2+^ from the SR by fine-tuning the activity of RyR2. Pathological perturbations in Zn^2+^ homoeostasis will lead to SR Ca^2+^ leak through irregular RyR2 channel openings but also increased activity of MG23. These data reveal that RyR2 is not the only ion channel contributing toward SR Ca^2+^ leak and suggest a new and important role for Zn^2+^ in regulating Ca^2+^ homeostasis and cardiac function.

## Experimental procedures

### Reagents

Chemicals were analar or the best equivalent grade from BDH (Poole, UK) or Sigma. All solutions were made in de-ionized water, and those for use in bilayer experiments were filtered through a Millipore membrane filter (0.45-μm pore). BAPTA, ATP, and the catalytic subunits of PKA were obtained from Sigma.

### Mg-23 knock-out mice

*Mg23* knock-out mice were produced using J1 embryonic stem (ES) cells derived from 129/Sv mice as described previously (60, 61). Briefly, genomic DNA fragments containing the *Mg23* gene were cloned from a mouse genomic library to construct the targeting vector in which the neomycin-resistance gene and the diphtheria toxin gene provide positive and negative selections, respectively. ES cells were transfected with the linearized vector and selected in a G418-containing medium. Several clones carrying the expected homologous mutation were isolated by Southern blot-screening of G418-resistant cells. The founder chimeric mice were generated using the positive ES clones and crossed with female C57B6/6J mice to produce heterozygous mutant mice. The genetic background of the *Mg23* knock-out mice resulting from crossing the heterozygous mice was 50:50 129/Sv:C57B6/6J. After back-crossing these mice more than 6 times with C57BL/6J mice, the *Mg23* knock-out and wild-type littermates (17–29 weeks old) used for this study were obtained. To confirm the mouse genotypes, PCR analysis was conducted using the MG23F and MG23R primers as described previously (60).

The institutional ethics committee at the University of St. Andrews and the University of Kyoto approved the study. Mice were killed by concussion followed by cervical dislocation. The care and sacrifice of the animals conformed to the guidelines from Directive 2010/63/EU of the European Parliament on the protection of animals used for scientific purposes. Full methods are detailed in the supplemental material.

### Single channel recording and analysis

Sheep cardiac heavy sarcoplasmic reticulum (HSR) membrane vesicles were prepared and fused with planar phosphatidylethanolamine lipid bilayers, as described previously (15). Sheep hearts were obtained from a local abattoir that conforms to the European Union directive 1099/2009 legislation. HSR vesicles from wild-type and *Mg23* knock-out mice were prepared in an identical fashion to sheep HSR vesicles, with the exception that 10 complete mouse hearts were used per membrane preparation. Single channel current recordings were monitored under voltage-clamp conditions using a BC-525C amplifier (Warner Instruments, Harvard) using previously described techniques (15). The *trans* chamber was perfused with 250 mm glutamic acid and 10 mm HEPES (pH 7.2) with Ca(OH)_2_ (concentration of free Ca^2+^ ∼50 mm). The identity of RyR2 was confirmed by the single-channel conductance, and the number of channels gating in the bilayer was assessed by the application of 500 μm suramin at the end of the experiment. The average capacitance of our bilayers was 89 ± 8 picofarads, and this was consistent throughout the entire recording. Experiments were carried out at room temperature (22 ± 2 °C). The concentration of free Ca^2+^ and pH of all of our solutions was determined using a Ca^2+^ electrode and a pH electrode (Hanna Instruments, Bedfordshire, UK) as previously described (15). Zn^2+^ was added as ZnCl_2_ to the *cis* chamber at the required concentration from an appropriate stock solution as described previously (15).

Measurements of current amplitude were made using the WinEDR 3.6.4 program. The closed and open current levels for both RyR2 and MG23 were assessed manually using cursors. All-points-amplitude histograms were obtained from single-channel data and constructed using QuB software Histograms of these distributions were fitted with mixtures of Gaussian curves using OriginPro software (OriginLab). Channel events were detected by the 50% threshold method (62) using TAC 4.2.0 software (Bruxton Corp.). Open probability (*P*_o_) was taken from 3 min of continuous recording. As reported previously, multiple MG23s often gated together in the bilayer (8). When many channels incorporated into the bilayer, we used noise analysis to estimate channel activity and to observe the effects of Zn^2+^ on channel function. Noise analyses of MG23 experiments were performed using WinEDR 3.6.4 as described previously (63).

### Affinity purification of Mitsugumin 23

MG23 channels were enriched and purified from sheep cardiac HSR vesicles. HSR vesicles were solubilized for 60 min at 4 °C at a protein concentration of 2 mg/ml in a CHAPS detergent solubilization buffer containing 1% w/v CHAPS, 1 m NaCl, 0.1 mm EGTA, 0.15 mm CaCl_2_, 25 mm PIPES, and 2.5 mm DTT. Insoluble material was removed by centrifugation for 30 min at 150,000 × *g*. The supernatant was incubated for 120 min with protein G-Sepharose (GE Healthcare) cross-linked with an αTMEM109 antibody (Sigma). The resin was washed in 20 mm Tris·HCl, 500 mm NaCl, 0.1% CHAPS, protease inhibitor mixture P8340 (Sigma) (1:500), 1 mm PMSF, 2.5 mm DTT (pH 7.4). MG23 protein was recovered by overnight incubation with an epitope peptide (Genscript) at 4 °C. Epitope peptide was removed by gel filtration using a HiTrap desalting column (GE healthcare). Elution fractions were collected. Identification of MG23 within each fraction was determined by sodium dodecylsulfate (SDS)-PAGE followed by silver staining. MG23-positive fractions were snap-frozen and stored at −80 °C.

### Measurement of free metal ion levels

The free Ca^2+^ concentration of our recording solutions was reduced from ∼5 μm to 100 nm by the addition of 7.6 μm concentrations of the chelation agent BAPTA. The free Ca^2+^ concentration was measured using a Ca^2+^-selective electrode. BAPTA is known to bind other divalent cations including Zn^2+^ (64). We, therefore, calculated the theoretical concentration of total Zn^2+^ required to be added to our low Ca^2+^ recording solutions in order to result in the appropriate free Zn^2+^ concentration. Free Zn^2+^ calculations were performed using the MIQUV program. Tris protonation and Zn^2+^ stability constants were taken from Bologni *et al.* (65). Calculations were performed using the ionic composition of TRIS/HEPES recording solutions at room temperature (22 ± 2 °C). Calculations assumed a starting free Ca^2+^ of 3.54 μm and a starting free Zn^2+^ of 9 pm. Free Ca^2+^ and Zn^2+^ were calculated in the presence of 7.6 μm BAPTA and following the incremental increase of Zn^2+^ concentration in the range 100 pm to 10 μm. *K_d_* values of BAPTA for Zn^2+^ and Ca^2+^ were based on previously reported values (64, 66). The calculated values are shown in supplemental Table S1.

The addition of ZnCl_2_ up to a concentration of 10 μm to our BAPTA-treated solutions had no significant effect on either the measured or the calculated free Ca^2+^ concentration (supplemental Fig. S2 and Table S1). Detection of free Zn^2+^ was performed using a pZn meter as described previously (15) and determined to be 9 ± 7 pm.

### Cell culture

H9C2 cells were cultured in Dulbecco's modified Eagle's medium (DMEM) supplemented with 10% fetal bovine serum and 1% penicillin/streptomycin (Life Technologies) under 95% air, 5% CO_2_ and sub-cultured between 60 and 70% confluence.

### H9C2 hypoxia

H9C2 cells were subjected to 0.5% O_2_ in a Don Whitley H35 hypoxystation (Don Whitley Scientific Ltd., Shipley, UK). Under hypoxic conditions cell lysates were collected in radioimmunoprecipitation assay buffer (25 mm Tris·HCl, 150 mm NaCl, 1% Nonidet P-40, 1% sodium deoxycholate, and 0.1% SDS supplemented with protease inhibitor mixture P8340 (Sigma). Lysates were snap-frozen and stored at −80 °C before use.

### Cellular Zn^2+^ measurements in H9C2 cells in response to ischemia

To simulate ischemia, H9C2 cells were exposed to an ischemia buffer containing 108 mm NaCl, 16 mm KCl, 0.33 mm NaH_2_PO_4_, 10 mm PIPES, 20 mm deoxyglucose, 20 mm sodium lactate, 1 mm MgCl_2_, 2 mm CaCl_2_, and 0.2 mm 2,4-dinitrophenol, pH 6.8, with NaOH. After aspiration of ischemia, buffer cells were washed in phosphate-buffered saline (PBS) and incubated with 5 μm ZnAF-2 (Chemodex, St. Gallen, Switzerland) in PBS in the dark for 30 min at 37 °C. Cells were subsequently washed in fresh PBS followed by 100 μm EDTA in PBS before fixing in 4% paraformaldehyde for 10 min at room temperature. Cells were then washed three times in PBS. Cells were permeabilized in 0.2% Triton X-100 in PBS for 30 min before loading with 10 μm 4′,6-diamidino-2-phenylindole (DAPI) in PBS for 10 min at room temperature. Basal Zn^2+^ fluorescence intensity (excitation 492 nm; emission 514 nm) was determined by FLUOstar OPTIMA microplate reader (BMG Labtech) and was normalized to DAPI (Life Technologies; excitation 358 nm; emission 461 nm).

### Western blot analysis

HSR proteins or H9C2 cell lysates were size-fractionated by SDS-PAGE on a 4–12% Bis-Tris precast gel (Life Technologies). After separation, proteins were transferred to a nitrocellulose membrane, and nonspecific binding sites were blocked for 1 h at room temperature using 5% dried milk and Tris-buffered saline (0.1% Tween 20, pH 7.4). Membranes were probed overnight at 4 °C with primary antibodies specific for RyR2 phosphorylated at site serine 2809 (1:5000, Badrilla, Leeds, UK), anti-MG23 (1:20,000 Sigma), and anti-β-actin (1:10,000, Sigma) in 5% dried milk and Tris-buffered saline. Secondary horseradish peroxidase-linked goat anti-rabbit or anti-mouse IgG (both 1:10,000) Sigma) antibodies were used in combination with an enhanced chemiluminescent detection system (Thermo Scientific, Paisley, UK) and visualized using a Fujifilm LAS-3000 detection system. Densitometry was performed using ImageJ software (National Institutes of Health).

### Statistical analyses

Data are shown as the mean ± S.D. Where appropriate, Student's *t* test was used to assess the difference between mean values. Where multiple treatments were compared, a one-way analysis of variance (ANOVA) followed by a Bonferroni post hoc test was used to assess the difference between treatments. *p* values ≤0.05 were taken as significant.

## Author contributions

B. R.-O. and G. B. R. designed and performed the experiments, analyzed the data, and contributed to writing the manuscript. A. K. and C. M. performed the experiments and analyzed the data. W. B. provided the theoretical calculations of free ions. H. T. and M. N. designed the experiments and provided key experimental tools. A. J. S. designed the experiments and contributed to writing the manuscript. S. J. P. designed the experiments, analyzed the data, wrote the manuscript, and supervised the project. All authors discussed results and commented on the manuscript at all stages.

## Supplementary Material

Supplemental Data

## Footnotes

4 The abbreviations used are:

RyR2: type-2 ryanodine receptor
MG23: mitsugumin 23
EC coupling: excitation-contraction coupling
SR: sarcoplasmic reticulum
HSR: heavy sarcoplasmic reticulum
*P*_o_: open probability
BAPTA: 2,2′-ethylenedioxy)dianiline-*N,N,N*′*,N*′-tetraacetic acid
DA: diacetate
ZIP: Zrt/Irt-like protein
FKBP: FK-506-binding protein
SERCA: sarco(endo)plasmic reticulum calcium ATPase
ES: embryonic stem
Bis-Tris: 2-[bis(2-hydroxyethyl)amino]-2-(hydroxymethyl)propane-1,3-diol
ANOVA: analysis of variance
ER: endoplasmic reticulum.
