# Supplementary material for: Dysregulated Zn2+ homeostasis impairs cardiac type-2 ryanodine receptor and mitsugumin 23 functions, leading to sarcoplasmic reticulum Ca2+ leakage
Source: J Biol Chem. 2017 Jun 19;292(32):13361–73. doi: 10.1074/jbc.M117.781708 (PMC5555195; doi:10.1074/jbc.M117.781708)
Supplement: Supplemental Data [file supp_292_32_13361__index.html]

Dysregulated Zn2+ homeostasis impairs cardiac type-2 ryanodine receptor and mitsugumin 23 functions, leading to sarcoplasmic reticulum Ca2+ leakage — Dysregulated Zn2+ homeostasis impairs cardiac type-2 ryanodine receptor and mitsugumin 23 functions, leading to sarcoplasmic reticulum Ca2+ leakage — Zn2+ and SR Ca2+leak — Supplemental Data 

# Dysregulated Zn2+ homeostasis impairs cardiac type-2 ryanodine receptor and mitsugumin 23 functions, leading to sarcoplasmic reticulum Ca2+ leakage

## Supplemental Data

- Supplemental Data (.pdf, 382 KB) - Supplemental data
