# Supplementary material for: Dysregulated Zn2+ homeostasis impairs cardiac type-2 ryanodine receptor and mitsugumin 23 functions, leading to sarcoplasmic reticulum Ca2+ leakage
Source: J Biol Chem. 2017 Jun 19;292(32):13361–73. doi: 10.1074/jbc.M117.781708 (PMC5555195; doi:10.1074/jbc.M117.781708)
Supplement: Supplemental Data [file 10.1074_M117.781708_jbc.M117.781708-1.pdf]

Dysregulated Zn^{2+} homeostasis impairs cardiac type-2 ryanodine receptor and mitsugumin 23 functions, leading to sarcoplasmic reticulum Ca^{2+} leakage

Benedict Reilly-O'Donnell*[1], Gavin B. Robertson*[1], Angela Karumbi[1], Connor McIntyre[1], Wojciech Bal[2], Miyuki Nishi[3], Hiroshi Takeshima[3], Alan J. Stewart[1] & Samantha J. Pitt[1#]

[1]School of Medicine, University of St Andrews, St Andrews, KY16 9TF, UK. [2] Department of Biophysics, Institute of Biochemistry and Biophysics, Polish Academy of Science, Warsaw, 02-106 Poland. [3]Graduate School of Pharmaceutical Sciences, Kyoto University, Kyoto, 606-8501, Japan.

Supplemental Data

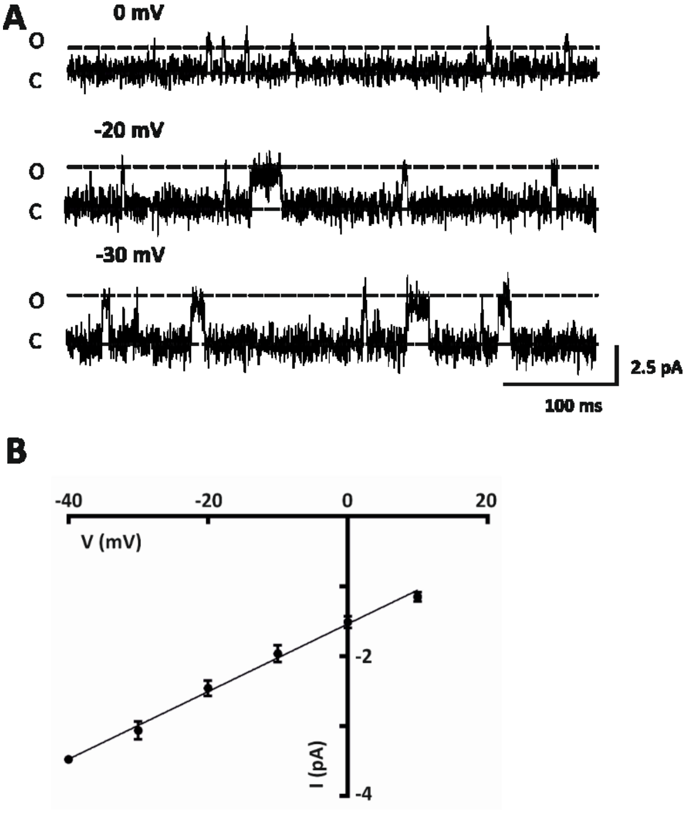

Figure S1. Single-channel properties of purified MG23. **(A)** Purified MG23 single-channel recordings using Ca^{2+} as the permeant ion at the indicated holding potential. The traces in this figure are not representative of Po but were chosen to show current amplitudes clearly. O and C show the fully open and closed states, respectively. **(B)** Current-voltage relationship of MG23 with Ca^{2+} as the permeant ion. Values are mean ± SD (n = 6).

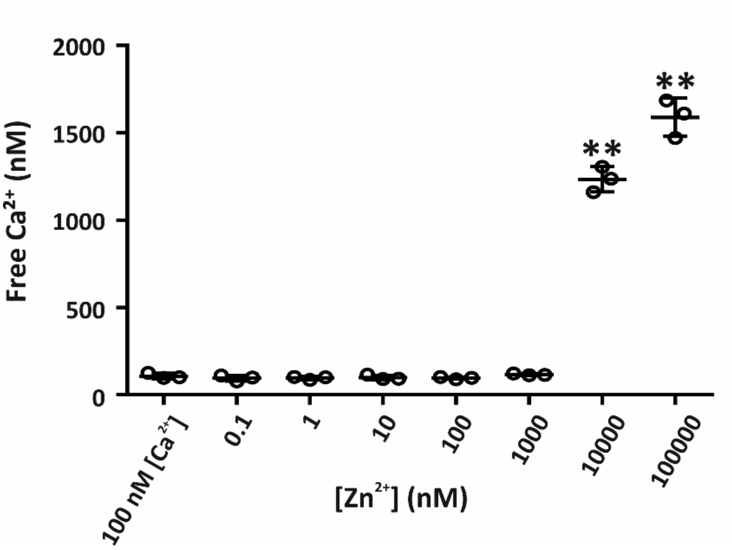

Figure S2. Effects of Zn^{2+} on Ca^{2+} levels in BAPTA treated recording solutions. The free [Ca^{2+}] of our TRIS/HEPES solution prior to the addition of BAPTA was determined using a Ca^{2+}- selective electrode. In order to lower the [Ca^{2+}] of our TRIS/HEPES solution, BAPTA was added until the measured free [Ca^{2+}] was 100 nM. Incremental increase of Zn^{2+} in the range 100 pM - 100 µM was added as $ZnCl_2$ to the BAPTA-treated solution and shown to have no significant effect until the [Zn^{2+}] ≥ 10 µM. Data are displayed as mean ± SD, *n*= 3. (**) indicates significance ($p \leq 0.01$) when compared to 100 nM [Ca^{2+}].

Calculated free [Zn^{2+}] and [Ca^{2+}] in BAPTA-treated TRIS/HEPES solutions		
Total [Zn^{2+}] (nM)	Free [Zn^{2+}] (nM)	Free [Ca^{2+}] (nM)
0.109	0.000204	91.3
1.009	0.0019	91.4
10	0.0188	91.6
50	0.0946	92.4
100	0.192	93.5
1000	2.43	118
10000	1290	2600

Table S1: Calculated free [Zn^{2+}] and [Ca^{2+}] in BAPTA-treated TRIS/HEPES solutions. Free Zn^{2+} calculations were performed using the MIQUV program. Calculations were performed using the ionic composition of TRIS/HEPES recording solutions at room temperature (22 ± 2 ºC).

References

1. Bologni, L., Sabatini, A., and Vacca, A. (1983) Complex formation equilibria between 2-amino-2(hydroxymethyl)-1,3,-propanediol (tris, tham) and nickel(II), copper(II), zinc(II) and hydrogen ions in aqueous solutions. *Inorg Chimica Acta* **69**, 71-75
